# Agoraphobia

**Published:** 1885-09

**Authors:** 


### Editorial.

### AGORAPHOBIA.

It has been truly said that man is conditional by his environment. The exciting conditions of modern life produce a wear and tear on nerve tissue, or, perhaps, more properly speaking, an irritability of the nerve centers, manifesting itself by irregular action in many directions. Of the various forms of nervous disorders which *seem* to be the products of the activity of present social conditions, and which were unknown to, or at any rate not mentioned by, the observers in medicine of the past generation, perhaps none is more singular or inexplicable than that now called agoraphobia; literally, fear of the "market-place" — like most others, an imperfect term to describe a varying condition, but sufficiently accurate for the purpose intended.

Fortunately for the sufferers, the disease is rare, although, to some, temporary attacks are sufficiently frequent to enable them to comprehend the sensations when more fully developed and permanent in others. As it is difficult to present any picture to our imagination, unless we have some pre-conceived standard for comparison, it seems well nigh impossible to convey in language any intelligible idea of the peculiar sensations experienced by sufferers from this disorder. It is said only to manifest itself in adults of education, and always in those otherwise devoid of fear; and while the essence of the disease seems to be *terror* without *real* cause, it is by no means akin to cowardice in the ordinary sense.

Conscious of this weakness, and the possibility of being misunderstood, the sufferer is always anxious to conceal the evidences of his infirmity.

A most typical instance came under the observation of the writer, occurring in a man of about 40 years of age, of good habits, physically well developed, and possessed of at least an ordinary degree of courage. The first attack happened while driving a particularly gentle horse, to which he was well accustomed. As he described it, he was overcome with an awful feeling of *dread*, accompanied by a consciousness of utter inability to guide the horse. Again, the idea of being in the room with a second story window open produced this feeling of terror. The suggestion of going near the brow of a moderate hill — *not the actual performance*, but the bare suggestion of such a course — would bring on an attack, amounting to almost abject terror, giving him the appearance of having received a violent shock. Again, the possibility of having to cross a bridge, even with a high wall on each side, brought on an attack of suspension of will power — motor impotence. At times, to go up and down stairs required an agonizing effort of will.

Nothing would induce him to go into the gallery of a theater, church, or other large edifice; and in all this there was not the slightest feeling of *fear* in its ordinary sense — that is, fear of bodily injury — but an indescribable feeling of dread, and a sort of isolation from all surroundings. The ever-present consciousness of this disease was a great source of mortification to him, and every effort to overcome the feeling was made, but without effect. He attributed the origin of the trouble to an attack of insolation, and to exhausting mental labor during convalescence. There was, also, in this case, some impairment of tactile sense, and a consciousness of his body being divided at the median line. An attack of hemiplegia, with frequent attacks of hemicrania, showed serious neurotic disturbance, but without any apparent ill health. He complained of being easily fatigued by slight mental or physical effort. Exposure to the sun's rays almost invariably brought on lateral headache and prostration. Here there seemed to be a direct cause, namely the *insolation.*

In other cases, again, the disease can be traced to no definite cause, and only one condition excites an attack, and that invariably; for instance, there is a case related of a gentleman in New York, in apparently good health, who cannot leave streets with stores in them.

A recent case is also related of a Russian colonel who suffered when in crowded streets, but who could ride at the head of his regiment, through the same thoroughfares, with impunity.

In the case above related in detail, the patient could cross a bridge by fixing his eye on some object and crossing as rapidly as possible. To stop, or have any one in front of him, brought on at once this nervous attack.

Many theories are advanced to account for the disease, none of them satisfactory in the present state of our ignorance of the nervous system. Over-work, gastric disturbances, the gouty diathesis, have been credited with the cause; in short, any thing which tends to exhaust nerve force pre-disposes to an attack. It is uncertain whether it is purely physical or psychical in origin.

It is more than probable, however, that the disease is cerebral, and caused by some slight organic changes in the brain.

Drugs seem to have but little effect; a complete change of surroundings and habits will most conduce to amelioration of the symptoms. In marked cases, and in those past middle life, there is not much prospect of radical cure. The best that can be hoped for is the avoidance of circumstances that excite the disease, and the improvement of the general health.

The subject is full of interest to the student of neural pathology, and calls for more extended study and investigation than it has yet received.

---

In the publication of the JOURNAL for the past year, our editorial labors have been materially lessened through assistance generously tendered by several able collaborators. The pressure of professional work rendered such assistance absolutely necessary in order to maintain the high standing and character of the JOURNAL. In the August numbeı, we intimated that the aid of collaborators was assured for this volume. It is fair, inasmuch as it has become necessary to seek aid in our work, that full recognition should be made and credit given to whom it is due.

While, during the present year, the proprietorship of the JOURNAL will remain with the senior editors, and its professional policy be directed by them, they will be assisted in the editorial work by the associate editors, Drs. Floyd S. Crego, Carlton C. Frederick, F. R. Campbell and Herbert Mickle. The names of these accomplished gentlemen are a sufficient guarantee of the character of the work they will perform for the JOURNAL. The senior editors are anxious to be relieved from the extra labor heretofore imposed, and have, therefore, called to their aid young men, full of energy and zeal, who will infuse new life and greater professional interest into journalistic work.

The past year has witnessed the most successful period in the history of the JOURNAL. We have had a larger circulation and have given a more able periodical. The present volume, of which the September issue is the second number, invigorated by the zeal and enthusiasm of our scholarly associates, cannot fail to equal, and, indeed, surpass in professional interest and value any of our monthly contemporaries. We may expect, therefore, increased success under the new regime.

---

Dr. Frank H. Potter, lecturer on materia medica in the Niagara Medical College, has sailed for Europe, to be absent for several months, which will be devoted to study and professional observation and improvement. Dr. Potter has been a faithful collaborator in the JOURNAL, and an ever-ready assistant in our work. The advantages of foreign travel and study will supplement the excellent preparation for his life-work in medicine, which he has already acquired. An earnest student, he will find in the great medical centers of Europe opportunities which will be faithfully improved. We tender our best wishes for success and health, and a happy return.

---

Notwithstanding all that has been written and said, within the past few years, upon the careful treatment of wounds, accidental and operative, it seems, as yet, beyond the comprehension of some medical men that there has been, in this direction, a *real advance*.

At the meeting of the American Medical Association in 1884, Prof. Dennis of New York read a paper giving the results of 144 compound fractures treated in Bellevue Hospital. No deaths occurred from septic infection, and in 100 cases of the 144, no death from any cause.

Again, turn to Volkmann's Statistics (Halle, 1882): 130 amputations for injury, 24 deaths, mortality 19 per cent.; 187 amputations for *disease*, with 7 deaths, mortality 3 per cent. And this in a hospital where Volkmann himself said, a few years ago, and before the introduction of anti-septic methods, that he was afraid to open a peritoneum, for fear of septicæmia and death, so great had been the mortality following even minor operations.

